# Cultural differences in the efficacy of unexpected questions, sketching, and timeline methods in eliciting cues to deception

**DOI:** 10.3389/fpsyg.2023.1175333

**Published:** 2023-08-31

**Authors:** Irina Tache, Lara Warmelink, Paul Taylor, Lorraine Hope

**Affiliations:** ^1^Department of Psychology, Lancaster University, Lancaster, United Kingdom; ^2^Department of Psychology, University of Portsmouth, Portsmouth, United Kingdom

**Keywords:** deception, cultural differences, individualism and collectivism, deception cues, LIWC

## Abstract

Asking unexpected questions, asking the interviewee to sketch the room, and asking the interviewee to make a timeline are techniques that have been shown to help an interviewer detect deceit. However, evidence of the efficacy of these techniques comes from studies of North American and North-West European participants, who are on average more individualistic (i.e., value individual achievements and uniqueness over group achievements) than people from other parts of the world. In two experiments involving participants with individualistic and collectivistic cultural backgrounds, we provide a more culturally diverse test of these techniques. Specifically, this study describes two experiments that investigated these interviewing techniques with people who are recent migrants to the UK. Experiment 1 used the LIWC categories “I,” “we,” “cognitive processes,” and “social processes” as the dependent variables; Experiment 2 measured details provided in a sketch and a timeline. The results show no effects of veracity in either of these experiments, although various effects of cultural differences in the outcome variables were observed. This suggests that cues to deception may not necessarily generalize to people from different cultural backgrounds. These results highlight the importance of conducting lie detection research across different countries and cultures.

## Introduction

The amount of information an interviewee reports, particularly when operationalized as the number of details provided, has been shown to be a cue to deception (DePaulo et al., 2003). However, this cue is often weak: The effect size is small and can be highly dependent on context (Luke, 2019). In response to this concern, researchers have developed techniques that elicit more and/or different details from interviewees. The increase in information is valuable in its own right in applied contexts, such as police interviews (Memon et al., 2010), and it can also increase the difference between truth-tellers and liars in the amount and type of information they provide, thus improving discrimination (Vrij and Granhag, 2012).

Asking unexpected questions has been shown to increase the capacity to identify deception about past events (Vrij et al., 2009; Lancaster et al., 2013) and future intentions (Warmelink et al., 2012, 2013; Sooniste et al., 2013). This may be because liars prepare “cover stories” by anticipating what an interviewer might ask (Clemens et al., 2013); thus, asking unexpected questions forces liars to create spontaneous lies. Coming up with a spontaneous lie that is credible and/or plausible likely generates additional mental load for the liar. By contrast, truth-tellers can rely on their memory to answer both expected and unexpected questions, so they are not negatively impacted by unexpected questions. Similarly, making sketches during the interview can help interviewees provide more details in two ways: They can provide details in the sketch itself (Vrij et al., 2010), or the act of producing the sketch may help them remember and verbally report more details (Deeb et al., 2021). In both cases, truth-tellers typically provide more detail than liars, and so the relative absence of details is a cue to deception. Finally, timelines are similar to sketches: Interviewees are asked to provide their accounts using a physical timeline to link events, people, and actions. In the context of truthful witnesses, the timeline technique helps interviewees provide more information (cf. control interviews; Hope et al., 2013, 2013). The technique has also been used with pairs of truthful or deceptive participants (Jundi et al., 2013). They found that truth-telling pairs asked each other more questions whilst building the timeline and that pairs could be accurately classified as truthful (71% correct) or deceptive (87% correct) based on the timeline task.

One major limitation of the deception literature is that the majority of research has been conducted in the US and the UK: 39% of deception studies originate from the US and 11% from the UK, whilst almost 7% of deception studies emerged from Canadian labs (Dineault et al., 2022). This regional profile is particularly concerning as research has shown that cues to deception differ between populations from different countries (Taylor et al., 2014; Leal et al., 2018). For example, in Leal et al. (2018), although truth-tellers provided more details than liars across all cultural groups in the study, British participants provided more visual, spatial, and action details than Arab and Chinese participants. Similarly, Taylor et al. (2014) found that white British truth-tellers provide more contextual details compared to white British liars, whilst Pakistani liars tended to provide more such details compared to Pakistani truth-tellers, inverting the cue to deception. Vrij and Vrij (2020) also found that Russian, Hispanic, and Korean samples differed in the cues to deception they provided: For Korean and Hispanic samples, the total number of details provided was a cue to deception (with a small effect size), whilst for a Russian sample, detail level was not a cue to deception. Tabata and Vrij (2023) research investigated the use of verbal strategies in a sample of Japanese adults. They found that, although several reported deception strategies in this sample matched strategies reported in the literature by participants from Western countries, there were also strategies reported by Japanese participants that did not occur in the Western samples in the literature. These differences in culture between populations from different countries may be a consequence of differences between these populations.

There are several distinct types of cultural differences between populations in different countries. Hofstede and Bond (1984) identified four: individualism–collectivism; power distance; uncertainty avoidance; and masculinity-femininity. Despite its complexity, individualism–collectivism is one of the most commonly used methods to compare cultures, and its relationship with a very wide range of behaviors has been studied (Fiske, 2002). Despite its commonness in the literature, or perhaps because of that commonness, there have been critiques of the value of individualism–collectivism amongst researchers (Hope et al., 2022). Individualism–collectivism is intended to measure the extent to which a culture values the individual over the in-group members or vice versa. Individualist cultures value concerns for individuals themselves and their immediate family, whilst in collectivist cultures, the in-group is more important, and members are expected to value and support the group as a whole (Hofstede and Bond, 1984). Individualism is associated with Western countries (such as the US and the UK), whilst collectivism is associated with Eastern and Southern cultures (e.g., China, Burkina Faso). However, this association between countries and individualism–collectivism creates a mismatch between individualism/collectivism at the level of countries and individualism–collectivism at the level of the individual (Hope et al., 2022). Even if we accept that there are differences in average (and see Oyserman et al., 2002 for some indication that these differences are smaller than expected), how do those differences translate to individuals or to individuals in varying contexts?

Despite this, differences in individualism–collectivism are associated with a wide range of behaviors and cognitions (Fiske, 2002), including communication styles, self-construal, and values (Gudykunst et al., 1996). Individualism–collectivism is not a single axis of differences: Individualism and collectivism can be expressed in a variety of ways. One of the most studied dimensions within individualism–collectivism is the extent to which a culture focusses on horizontal or vertical relationships, i.e., the extent to which a culture is hierarchical (Triandis and Gelfand, 1998). Whereas people with a greater horizontal individualistic focus emphasize the importance of being unique, someone with a more vertical individualistic focus will emphasize being the best. Similarly, horizontal collectivists tend to focus on the homogeneity and interdependence of the in-group, whilst vertical collectivists tend to emphasize sacrifice for the group and competition by the group against out-groups (Triandis and Gelfand, 1998). Taylor et al. (2014) suggest that differences in cues to deception between populations from different countries may be explained by cultural differences in individualism–collectivism because individualism–collectivism affects self-construal. Self-construal is the way in which people cognitively, emotionally, and behaviorally relate themselves to (and separate themselves from) others, and it is affected by cultural differences (Singelis and Sharkey, 1995). Taylor et al. (2017) showed that participants from collectivist cultures differed significantly from participants from individualistic cultures in how they changed their pronoun use when lying compared to when they were telling the truth. Where participants from a more collectivistic culture used first-person pronouns more in lies and third-person pronouns less in lies (compared to truths), participants from individualistic cultures used first-person pronouns less in lies and third-person pronouns more in lies (compared to truths). Taylor et al. (2017) suggest that this may be strategic: People from collectivistic cultures maybe attempting to disassociate in-group members from their lies to protect them, whilst people from individualistic cultures are more focused on disassociating themselves from their lie.

Other cultural dimensions likely also affect cultural differences in deception behaviors. For example, Leal et al. (2018) studied UK, Arab, and Chinese populations because these cultures differ in whether they are low communication context or high communication context cultures. People from high-context cultures rely more heavily on the context surrounding communication (e.g., background knowledge and body language) than people from low-context cultures, who tend to put more information in the communication itself. Tabata and Vrij focused on a Japanese sample, as Japan is a high-context culture, in contrast to the countries that have been extensively studied in deception research. However, differences between low and high-context cultures are linked to differences in individualism–collectivism (Gudykunst et al., 1996), which leads to the possibility that the differences in deception behavior between people from different cultures could be due to either dimension.

In the current study, the focus is on individualism–collectivism including its vertical and horizontal aspects because individualism–collectivism is one of the primary measures of cultural differences, and data on how it affects the cues to deception under investigation are available in the scientific literature. However, much of the available literature focusses on individualism–collectivism only; less information is available with regard to sub-divisions of individualism–collectivism.

In the current study, we report two experiments in which the effects of horizontal and vertical individualism and collectivism on cues to deception were investigated. The first experiment investigated these effects in interviews with expected and unexpected questions, whilst the second experiment examined these effects in interviews that included a sketch and a timeline. Both experiments recruited participants with diverse cultural backgrounds who were currently living in the UK.

## Experiment 1

In Experiment 1, participants with individualist and collectivist cultural backgrounds were asked to either lie or tell the truth about a future intention in the context of an interpersonal interview. All participants spoke English as a second language and were interviewed in English by British interviewers. A population consisting of non-native speakers was selected because being a non-native speaker affects lie detection. Specifically, non-native speakers report a lower ability than native speakers to control cues for deception (Cheng and Broadhurst, 2005). Observers of non-native speakers tend to show a lie bias (a tendency to report that the person is lying even when they are telling the truth), which is not present when observing native speakers. This may be due to stereotypes surrounding non-native accents (Wylie et al., 2022). Therefore, recruiting a mix of native and non-native speakers would have added a confounding variable (see Discussion).

To measure participants' individual cultural values (rather than relying solely on country-level data on cultural values), participants completed the Culture Orientation Scale (COS; Triandis and Gelfand, 1998). To ensure that the interview covered a broad range of questions, participants were questioned using expected and unexpected questions designed in a pilot study to be relevant to their cultural background (see Method).

In this experiment, we focused on three categories of Linguistics Inquiry and Word Count (LIWC) as cues to deception. LIWC is a piece of language analysis software that has been used in the field of deception for several decades (see, e.g., Newman et al., 2003). All three LIWC measures used are pertinent to both deception and individualism–collectivism. The first type of cues examined in this study was personal pronouns (e.g., “I” vs. “we”), which are affected by both individualism–collectivism and veracity. Specifically, research suggests that individualism is associated with the use of fewer “we” pronouns in an auto-photographic essay (Burke and Dollinger, 2005). With respect to veracity and the use of personal pronouns, Newman et al. (2003) found that deception is associated with first-person pronoun use: Liars use “I” less often than truth-tellers. Taylor et al. (2017) found that this reduction in the use of I (and a commensurate increase in the use of third-person pronouns) was only present in a sample of participants from individualistic countries. People from collectivistic countries showed the opposite effect.

The second cue examined was cognitive processes, a LIWC category that captures words that indicate speakers' cognitive processes surrounding the topic they are discussing (e.g., causation, differentiation, and insight). The use of these words is affected by both individualism–collectivism and veracity. Higher individualism is associated with more use of cognitive process words, likely because cognitive process words are associated with greater variety in individual expression (Burke and Dollinger, 2005). Individual expression is valued by people high in individualism, particularly horizontal individualism. Truth-telling is associated with the use of fewer cognitive processing words than those who are lying (Chiranjeevi et al., 2018).

The third cue examined was social processes, a LIWC category that covers words related to social behavior and social referents (e.g., conflict or family). Collectivism is associated with more use of social process words than individualism, likely because collectivists place greater value on social connectedness (Burke and Dollinger, 2005). Truth-telling is also associated with more social process words than lying (Chiranjeevi et al., 2018).

In light of these previous findings, we predicted that as the use of first-person pronouns is affected by both culture and veracity, individualists will use “we” less than collectivists (Hypothesis 1a) and liars will use “I” less than truth-tellers (Hypothesis 1b). We next predicted that both individualists and liars would use more cognitive processes than collectivists and truth-tellers (Hypotheses 2a and b). Finally, we predicted that individualists will use social process words less than collectivists, whilst liars will use fewer social process words than truth-tellers (Hypotheses 3 a and b).

## Method

### Participants

An *a priori* power analysis, using GPower and assuming an effect size (f) = 0.25, suggested that we would need 120 participants to achieve a power of 0.8, at an alpha level of 0.05. Participants were second-language English-speaking undergraduates (N = 132; 44 males, 88 females, M age = 22.76, SD = 4.59) recruited at Lancaster University in the UK and paid £3.50 for their time completing the study. Participants had been residents of the UK for an average of 2.23 years (SD = 1.45). When participants were recruited, they were classified as individualist or collectivist based on the individualism–collectivism score of their country of birth (see https://www.hofstede-insights.com/fi/product/compare-countries/), with scores below 50 leading to assignment as collectivist and scores over 50 to assignment as an individualist; participants whose Hofstede score was undetermined were excluded from the analysis. On the basis of this classification, the sample comprised 66 participants from countries that are collectivist in orientation and 59 participants from countries considered individualist in orientation (6 participants were unclassified due to individualism–collectivism scores not being available for their countries of birth). Participants in the individualist group had a mean Hofstede score of 67.00 (SD = 9.28), whilst those in the collectivist group had a mean Hofstede score of 27.16 (SD = 10.60). Participants' reported countries of birth were China (N = 22; Hofstede score = 20); France (N = 11; Hofstede score = 71); Germany (N = 10, Hofstede score = 67); Nigeria (N = 8; Hofstede score = 30); Italy (N = 7; Hofstede score = 76); Bulgaria (N = 7; Hofstede score = 30); Hong Kong (N = 6; Hofstede score = 25); Lithuania (N = 5); Spain, Poland, Hungary, India (N = 4); and 28 further countries.

Participants' language ability was assessed by their most recent University-approved English language tests (e.g., Cambridge CPE, IELTS) or, where this was not available (29% of participants), by their self-reported ability on a scale from 1 (very poor) to 7 (very good). Test results were mapped onto the 7-point scale. Participants' English-speaking ability was reported to be on average in the good to very good range (M = 5.89, SD = 0.76).

### Design

The study had a mixed design with veracity (between-subjects: truth vs. lie), culture (between-subjects: individualistic vs. collectivistic), and question expectedness (within-subjects: expected by all, unexpected by all), expected by individualists (i.e., more expected by individualistic participants than collectivistic), and expected by collectivists (i.e., more expected by collectivistic than by individualistic participants) as independent variables.

The dependent variables were the percentage of words in the participants' answers that were assigned by LIWC to the following LIWC categories: first-person pronouns, third-person pronouns, cognitive processes, and social processes.

### Materials

#### Interview questions

We took care to develop an interview protocol that had culturally appropriate expected and unexpected questions. In a pilot study, 29 undergraduates {M age = 32.97, SD age = 12.11; 14 individualists [mean Hofstede score = 85 (SD = 8.17)] and 15 collectivists [mean Hofstede score = 27 (SD = 5.68)]; 15 males, 14 females} were recruited via word of mouth at the same university as the main study. These participants did not take part in the main study. A set of 46 interview questions relating to the topic of the interview (travel to the participants' home country) were generated. Participants were asked to rate the expectedness of these 46 possible questions on a 4-point scale. This resulted in a list of questions separated into four categories, based on the cultural background of those who rated them: expected by all (e.g., “Tell me everything about your intention”), unexpected by all (e.g., “What was a difficult thing to plan for this intention?”), individualist-expected/collectivist unexpected (e.g., “Please describe how you feel about this trip”), and individualist-unexpected/collectivist-expected (e.g., “How will the people who you are going to see feel about your trip?”).

The final interview question list (12 items/questions, see Appendix 1) was developed by selecting questions that were rated most or least expected by everyone, and questions that were rated most expected by one culture whilst most unexpected by the other. The final question list began with a general question about the intention (the most expected question) and continued with specific questions about a particular aspect of the intention (most important aspect of intention; the most important part of the travel; the most important person). This question list was asked in the same order for all participants.

#### Post-experiment questionnaire

A post-interview captured participants' gender, age (in years), motivation (10-point scale), and preparation (10-point scale). To gain a better understanding of our sample and their cultural background, self-reported ethnicity, country of birth, country of permanent residence, current country of residence, and the date they moved to the United Kingdom were also recorded. As the Hofstede score measures culture at the country level and individuals may differ substantially from their countrymen's average, we also wanted a measure of individual cultural values. To measure their individualism–collectivism values, participants completed the Culture Orientation Scale (COS). The COS is a 16-item scale, with 4 subscales: horizontal individualism, vertical individualism, horizontal collectivism, and vertical collectivism (see Triandis and Gelfand, 1998 for all items and validity information). Horizontal individualism is associated with strong positive values toward independence (people high in HI endorse items such as “I'd rather depend on myself than others”), vertical individualism is associated with competition (endorsing items such as “Winning is everything”), horizontal collectivism is associated with cooperation (endorsing, e.g., “I feel good when I cooperate with others”), and vertical collectivism is associated with a strong connection with family (endorsing “family members should stick together, no matter what sacrifices are required”). As validation of our manipulations, participants also rated the questions' expectedness and the likelihood and familiarity of the event discussed (on a 10-point scale). They were also able to comment on their experience in an open text box.

### Procedure

Participants were met by the researcher and informed that they would be interviewed about a specific future intention: The next time they would travel to their home country. This event was chosen because of its relevance to all of the participants in the near future as they all moved to live in the UK for the duration of their studies. Participants in the “truth” condition were instructed to tell the truth about their intention. Participants in the “lie” condition were instructed to lie about what they intended to do and make sure that they do not share any details about what they are truthfully intending with the interviewer. They were not given any specific instructions on what that lie should be, except that it should be untrue. All participants were instructed to try to convince the interviewer of their truthfulness, and they knew the interviewer was expecting that some might lie.

After making sure the participants had understood the instructions and had consented to take part, they were given 10 min to prepare for the interview. After this time, participants were introduced to the interviewer. There were eight different interviewers, they were all native English-speaking, UK-based PhD students, and blind to the veracity condition of the participants and the hypotheses of this experiment [interviewers' mean scores for vertical collectivism = 27 (3.12), horizontal collectivism = 29 (4.84), vertical individualism = 18.13 (8.58), and horizontal individualism = 23.63 (3.89)]. The interviews were all recorded. Following the interview, participants completed the post-experiment questionnaires and were paid and debriefed.

### Analysis

Audio recordings of the interviews were transcribed, and the transcripts were analyzed using Linguistic Inquiry and Word Count (LIWC; Pennebaker et al., 2015). LIWC calculates the proportion of words in a text that match a set of over 90 categories that concern affective, cognitive, linguistic, and social dimensions. These categories have been shown to be both reliable (Tausczik and Pennebaker, 2010) and valuable in their contribution to the analysis of interviews (Richardson et al., 2014; Taylor et al., 2017).

### Modeling

Linear mixed effects models were run using R (R Development Core Team, 2015), through RStudio (RStudio Team, 2015), alongside the lme4 (Bates et al., 2015) and lmerTest (Kuznetsova et al., 2016) packages. Each dependent LIWC variable fit, in turn, to the same sequence of models: beginning with the (0) baseline model of random effects of participant and question number, adding to this the fixed effects of veracity, culture (as classified by Hofstede score), and question expectedness in (1) all main effects model, following this with an interaction effect of veracity and culture (2), and, finally, adding all main effects interactions model (3). It should be noted that, for all models, the random effects of participant and question type significantly accounted for some of the variance. As these effects are not themselves of interest, they are not described below.

Model comparisons were done between each complex model and its nested predecessor. The best-fit models were selected by observing the best agreement in the highest increase in the log likelihood ratio, given a significance check of a *p*-value of < 0.05 using a chi-square test. All models converged successfully.

## Results

### Manipulation checks

The COS did not consistently correlate with the Hofstede score (only one significant correlation: with horizontal collectivism: r = 0.20, *p* < 0.05, −0.09 < other COS scales r < −0.02, ns). Contrary to our expectations, we also did not replicate the expectedness ratings (see Table 1). There was no significant correlation between any of the COS measures and the expectedness ratings of any of the question types. We did find a small, but significant correlation (*r* = −0.2, *p* = 0.02) between the Hofstede score and questions expected by all: Participants from countries with more individualistic Hofstede scores rated the questions that in the pilot study were expected by participants from all countries as less expected than participants from countries with more collectivistic Hofstede scores.

**Table 1:** Correlations between measures of culture and measures of question expectedness.

	**All expected**	**All unexpected**	**Ind > col**	**Col > Ind**
Hofstede score	−0.20^*^	0.11	−0.16	0.08
Vertical collectivism	−0.07	−0.01	0.03	0.03
Horizontal collectivism	−0.03	0.14	0.01	−0.11
Vertical individualism	−0.09	−0.16	−0.09	0.04
Horizontal individualism	0.01	0.07	−0.07	−0.10

^*^indicate significance at a *p*-value of < 0.05 level.

The mean motivation ratings were high [7.72 out of a possible 10 (SD = 2.21)]. Liars reported a slightly higher motivation rating (M = 8.03, SD = 1.9) than truth-tellers (M = 7.4, SD = 2.46), but this is not a statistically significant difference [t_(130)_ = 1.74, *p* = 0.11]. Liars rated the likelihood of the event they discussed as less likely (M = 7.07, SD = 3.65) than truth-tellers [M = 8.58, SD = 2.52; t_(130)_ = 2.76, *p* = 0.01]. This indicates that participants understood and complied with the instruction to lie. However, some liars reported in the open text box that although the event that they discussed was in itself very likely, it was not an event that they intended to complete on their next trip. This suggests that the likelihood of the event is not a perfect proxy for veracity: i.e., some lies are very likely. Liars and truth-tellers did not significantly differ in the rating of their familiarity with the event they discussed [liars M = 8.21, SD = 2.45, truth-tellers M = 8.94, SD = 1.94, t_(130)_ = 1.98, *p* = 0.06]. This suggests that liars mostly choose to set their lies in familiar surroundings.

***Hypothesis 1: The use of first-person pronouns is affected by both culture and veracity*.** ***Individualists will use “we” less than collectivists. Liars will use “I” less than liars*. **

Model comparisons found that the random effects baseline model was the best fit for the word “I”. This suggests that veracity, culture, and question expectedness had no significant influence on its use. Instead, any difference found can be attributed to the random effect of either the participant or the question used.

Model comparisons showed the best-fit model for “We” pronoun use was the main effects model, with no interactions. The main effects model showed that contrary to the hypothesis, collectivists use fewer “We” pronouns (M = 0.59, SE = 0.06) than individualists (M= 0.84, SE = 0.06). There was no difference (F = 1.19, *p* > 0.32) between liars (M = 9.54, SE = 0.30) and truth-tellers (M = 9.99, SE = 0.30) or between different question expectedness (F = 3.79, *p* = 0.053) in the use of “We” pronouns.

***Hypothesis 2: Individualists and liars will use more cognitive processes than*** ***collectivists and truth-tellers*. **

Model comparisons showed that the three-way interactions model was the best fit for cognitive processing of words. In this model, the three-way interaction was not significant. Instead, there was a significant interaction between culture and question expectedness (F = 7.63, *p* < 0.001). To unpack this effect, the data were subset by question type, and *t-*tests were run between the two cultures. We found differences between the individualist and collectivist groups when answering expected [t_(344.35)_ = 2.79, *p* = 0.01] and unexpected by all [t_(355.62)_ = −2.33, *p* = 0.02] questions. Collectivists used more (M = 13.89, SD = 10.27) cognitive words when answering expected questions than individualists (M = 11.21, SD = 7.84) but fewer (M = 14.73, SD = 7.43) when answering unexpected by all questions compared to individualists (M = 16.58, SD = 7.54). There were no differences between the two cultures when answering individualist-expected [t_(364.45)_ = −1.72, *p* = 0.09] and collectivist-expected [t_(355.65)_ = 1.58, *p* = 0.11] questions.

***Hypothesis 3: Individualists will use social process words less than collectivists. Liars*** ***will use more social process words than truth-tellers*. **

Model comparisons showed the best-fit model of word use representing social processes was the main effects model, with no interactions. The culture effect was the cause of this model being better than the baseline model, although the culture effect itself is not significant (F = 3.84, *p* = 0.05): contrary to the hypothesis, collectivists used fewer words (M = 8.93, SE = 0.30) to represent social processes than individualists (M = 9.61, SE = 0.30). The main effects model showed that there was no difference (F = 2.39, *p* = 0.12) between liars (M = 9.54, SE = 0.30) and truth-tellers (M = 9.99, SE = 0.30) or between different question expectedness (F = 3.16, *p* = 0.08).

## Discussion

None of the hypotheses in this experiment were entirely supported. For Hypotheses 1b, 2b, and 3b, no effect of veracity was found using the LIWC categories I, we, cognitive processes, and social processes. For Hypotheses 1a and 3a, an effect of culture was found but in the opposite direction of the hypothesis. For Hypothesis 2a, an interaction effect between culture and question expectedness was found. The results also showed that our manipulation of question expectedness was not entirely successful: Culture did not affect the expectedness of questions in the way that was assumed based on the pilot. In fact, except for a small negative correlation between Hofstede Score and the expectedness of questions that in the pilot study were expected by all participants, there was no relationship between culture and question expectedness. This makes the interaction effect between culture and question expectedness difficult to interpret and means we cannot draw any strong conclusion on whether using unexpected questions as a way to elicit cues to deception is a technique that generalizes to non-Western cultures.

Taken together, these results suggest that the cues to deception previously identified in the literature were not present in this sample. Results from the individualism–collectivism literature also did not replicate. Although culture effects were present in this sample, they ran in the opposite direction of those reported in the literature for two hypotheses. This suggests a lack of generalization of the effects in the literature (Newman et al., 2003; Chiranjeevi et al., 2018) or a methodological issue in these comparisons (see General Discussion).

## Experiment 2

Experiment 2 investigated sketches and timelines as techniques to increase cues to deception in culturally diverse populations. Sketches have been shown to yield useful cues to deception. For example, Vrij et al. (2010) found that truth-tellers provided more plausible sketches, were more likely to include a Confederate in the drawing, and were more likely to use a shoulder-height point of view. Deeb et al. (2021) also found that sketches helped both truth-tellers and liars provide more core detail, although Vrij et al. (2022) results suggest that sketches may not benefit lie detection in online interviews. Although there is, to our knowledge, no direct research of how individualism–collectivism affects people's sketching in the context of information-gathering interviews, research suggests that individualists tend to be more focused on objects than collectivists, who tend to focus more on background fields (Gorodnichenko and Roland, 2012). This tendency may be reflected in people's sketches. The level of detail can be a cue to deception in timeline interviews [such as the adapted timeline format used by Izovotas et al. (2018)]. There has been little research into how culture affects timeline performance, although there is anecdotal evidence that people from non-Western cultures report less information compared to participants in studies conducted in Western countries (Hope et al., under review).

In Experiment 2, participants committed a mock crime that involved a Confederate using a scenario drawn from Vrij et al. (2010), who explored the use of sketching in interviews about a mission that involved a Confederate. In both Vrij et al. (2010) and Experiment 2, participants were then interviewed and asked to make a sketch of the location where they met the Confederate. In Experiment 2, participants were also asked to provide a timeline of their actions during the scenario.

Based on Vrij et al. (2010), the following hypotheses were formulated with respect to sketching: Truth-tellers will draw more objects (H1a), more people (H1b), and are more likely to draw the Confederate (H1c) than liars. Individualists will draw more objects (H1d) but fewer people (H1e) than collectivists. Truth-tellers are more likely to draw a shoulder camera position than liars, whilst liars are more likely to use an above-eye view position (H2a) than truth-tellers. Individualists are more likely to draw a shoulder camera position and less likely to use an above-eye position than collectivists (H2b).

For the timeline, the lack of previous data makes it harder to set clear evidence-based hypotheses specifically for the timeline. However, we assumed that timelines might show similar effects as interviews and sketches. Based on the literature on interviews (Luke, 2019) and sketches (Vrij et al., 2010), we hypothesized that truth-tellers would report more detail (object, people, and action) (H3) and that individualists would report more object detail (H4a), but fewer people detail (H4b) than collectivists.

## Methods

### Participants

As in Experiment 1, participants were living in the UK and were not native speakers of English. They were selected based on the Hofstede score of their country of birth. An *a priori* sample size analysis, using GPower assuming a large effect size (f = 0.4, α = 0.05, power = 0.8) recommended a total of 112 participants. A large effect size was assumed, based on strong effects reported by Vrij et al. (2010) and Jundi et al. (2013) for sketches and timelines, respectively. Originally 113 participants completed both the sketch and the timeline tasks in their interviews. The most common countries of birth were China and Italy (N = 10); India and Poland (N = 9), France (N = 6), Bulgaria, Indonesia, Malaysia, and Spain (N = 5) and 28 other countries. However, due to data loss at data collection (recording errors; Hofstede country data not available for some participants) and coding (loss of data in storage) not all participants' data were available for analysis. For clarity, the total samples are reported here for each task separately.

#### Sketches

For 17 of the 113 participants, no Hofstede score was available, due to their countries' data not being available, leaving 96 participants in the final sample. Of these participants, 59 reported being female, 36 male and one participant did not report their gender. The participants mean age was 23.30 (SD: 4.68). Fifty-two participants reported being white, 28 being East/South Asian/Pacific Islander, 7 other, 5 Hispanic/Latino, 2 Middle Eastern/Arab, and 2 black Caribbean/African/other.

#### Timeline

For 89 of the 113 participants, a coded timeline was available. Data loss occurred at the recording and coding stage, rather than the data collection phase. For 14 of those 89 participants, no Hofstede score was available, due to those participant countries' data not being available. This left 75 participants in the final sample for the timeline task. Of these 45 reported being females and 29 males, and 1 participant did not report their gender. Their mean age was 23.31 (SD = 4.36). Forty-one reported being white, 21 reported being East/South Asian/Pacific Islander, 5 other, 4 Hispanic/Latino, 2 Middle Eastern/Arab, and 1 black Caribbean/African/other.

### Design

The experiments had one independent variable: veracity (between subjects: truth-tellers vs. liars) and a quasi-IV: culture. As in Experiment 1, culture was measured in two ways: I) via the Hofstede score of the country of birth of the participants and II) via the COS scale. Unlike in Experiment 1, the Hofstede score and the four subscales of the COS were treated as five separate continuous variables for the analysis. This change was adopted after the findings of Experiment 1 showed that the correlation between these variables was lower than expected. The dependent variables include the number of details, objects, and people included in the sketch/timeline and the point of view in the sketch.

### Materials

For the sketching task, participants were provided with white, A4 paper, and a pencil. For the timeline task, participants were provided with a physical timeline made of a light card to act as the base of their timeline. They were also given a stack of post-it notes on which to write details of events to place on the timeline. Participants also completed the COS (Triandis and Gelfand, 1998). They were also asked to fill out a post-task questionnaire that contained a measure of their drawing ability, demographic details, measures of their motivation, and their experience of the task.

### Procedure

Participants came to the laboratory and were informed that they would be asked to take part in a scenario that might include actions that would be considered against university regulations, if they were done outside of the scenario. They were randomly allocated to the truth-telling or lying condition by selecting an envelope that contained condition-specific written instructions. All participants were instructed to go to a room in the library near the laboratory. Once there, they met with a person (who was a Confederate), requested a set of documents from this person, took these documents, left them in a prearranged location, and returned to the laboratory. All participants followed the same route. Participants in the truth-telling condition received instructions that they were helping the university by legitimately relocating a set of exam papers from a graduate teaching associate (the Confederate) to a safe location. Participants in the lying condition were told that they had been helping move exam papers for someone who stole them (the Confederate). When they returned to the laboratory, all participants were told that they were seen moving exam papers, that this was considered suspicious, and that they would be interviewed about this. Participants in the truth-telling condition were instructed to tell the truth about what happened. Participants in the lying condition were instructed to lie about what happened: In particular, they were told that they should not “give away” the person who they got the documents from. The interview consisted of sketching and timeline tasks. The order of these tasks was counterbalanced. They were instructed to sketch what happened when they received the documents and to make a timeline of all the events that happened from when they left the laboratory to when they returned. Participants were encouraged to verbally describe their thinking process as they completed these tasks, although this narration was not analyzed. After these tasks, participants were informed that the scenario had ended. They were asked to fill out the post-task questionnaire. They were then debriefed, received their reward, and thanked for their participation.

### Data analysis

Data were coded by the main experimenter (first author) and a reliability coder who was not otherwise involved with the project. The first coder coded 72% of the sketches and 62% of the timelines, and the second coder coded 52% of the sketches and 66% of the timelines. Sketches were coded for the number of objects (ICC = 0.89, 95% CI = 0.76–0.95), people (ICC = 0.80, 95% CI = 0.63–0.90), whether the Confederate was present (Cohen's kappa = 1), and the camera angle Cohen's kappa = 0.44, z = 4.37, *p* < 0.0001). The timeline was coded by the same coders for the number of each type of card that participants used and the objects, people, actions, and other details that they provided on each type of card (ICC = 1 for all types). The types of details were then summed across cards.

The data were analyzed using regression models in R. The independent variables were veracity, horizontal individualism, cultural individualism, horizontal collectivism, vertical collectivism, and Hofstede score. The dependent variable varied according to the hypothesis tested. For drawing, the Confederate and camera angle binary logistic regressions were run using the GLM function and family = binomial in R.

## Results

As in Experiment 1, the relationship between the participants' country of birth's Hofstede scores and their COS scores was low (all r's between −0.19 and −0.05). The two collectivism scales do correlate at 0.50; the two individualism scales at r = 0.35. Participants rated themselves as very seriously engaging in the task (liars M = 8.30, SD = 1.59, truth-tellers mean = 8.67, SD = 1.42) and highly motivated (liars M = 8.65, SD = 1.53, truth-tellers mean = 9.00, SD = 1.49) to convince the interviewer that they were telling the truth. These ratings did not differ between truth-tellers and liars [serious engagement: t_(109)_ = 1.29, *p* = 0.20; motivation: t_(109)_ = 1.22, *p* = 0.22].

### Sketches

***Hypothesis 1: Drawing objects, people, and the Confederate*. **Participants drew on average 6.89 objects (SD = 2.56), 0.39 people (SD = 1.17), and 90% included the Confederate. Vertical collectivism and horizontal individualism are associated with drawing fewer objects (VC estimate = −0.15, *t* = −3.30, *p* = 0.001; HI estimate = −0.12, *t* = −2.08, *p* = 0.04). There are no effects of veracity or any of the other culture scores (see Table 2). Neither culture nor veracity affected the number of people drawn or the Confederate (all t's between −1.21 and 1.23, all p's > 0.22).

**Table 2:** Coefficients of the independent variables on objects drawn in the sketch.

**Independent variable**	**Estimate**	**SE**	**t**	***p***
Intercept	13.51	2.33	5.80	< 0.001^*^
Veracity: Truth	0.12	0.51	0.24	0.81
Horizontal collectivism	−0.05	0.07	−0.75	0.45
Horizontal individualism	−0.12	0.06	−2.08	0.04^*^
Vertical collectivism	−0.15	0.05	−3.30	0.001^*^
Vertical individualism	0.05	0.04	1.25	0.21
Hofstede score	0.01	0.01	1.04	0.30

^*^indicates a *p*-value of < 0.05.

***Hypothesis 2: camera position*. **Neither veracity nor any of the culture measures affected camera position (all t's between −0.96 and 1.31, all p's > 0.23).

### Timeline reports

***Hypotheses 3 and 4*. **Higher scores on vertical collectivism were associated with reporting a lower number of object details (VC estimate = −0.23, *t* = −2.15, *p* = 0.04) and a lower number of person details (VC estimate = −0.86, *t* = −4.47, *p* ≤ 0.001) (see Table 3) than having lower scores on vertical collectivism. There was no effect of veracity or any other culture measure (all t's between −0.14 and 1.31, all p's > 0.23) on the number of action details (all t's between −1.00 and 0.19, all p's > 0.24).

**Table 3:** Coefficients of the independent variables on objects and people details reported in the timeline.

	**Objects**				**People**			

	**Estimate**	**Std. Error**	***t*** **-value**	**Pr(**>**|t|)**	**Estimate**	**Std. Error**	***t*** **-value**	**Pr(**>**|t|)**
Intercept	3.54	5.77	0.61	0.54	14.48	12.65	1.15	0.26
Veracity	0.04	1.19	0.04	0.97	2.12	2.78	0.76	0.45
Horizontal collectivism	0.16	0.17	0.97	0.34	0.36	0.39	0.93	0.36
Horizontal individualism	0.02	0.13	0.19	0.85	0.20	0.29	0.69	0.49
Vertical collectivism	−0.23	0.11	−2.15	0.04^*^	−0.86	0.25	−3.47	< 0.001^*^
Vertical individualism	0.06	0.10	0.64	0.52	0.18	0.23	0.78	0.44
Hofstede score	0.03	0.03	1.02	0.31	−0.01	0.06	−0.14	0.89

^*^indicates a *p*-value of < 0.05.

## Discussion

The results provide some support for Hypotheses 1d and 4a: Vertical collectivism was associated with providing less object detail in both the sketch and the timeline. This is in line with Gorodnichenko and Roland (2012) finding that individualism is associated with more object detail. However, contrary to Hypothesis 1d, high horizontal individualism was also associated with providing fewer object details in the sketch. Contrary to Hypothesis 4d, vertical collectivism was also associated with providing less detail about people in the timeline. In addition to these findings, the other hypotheses were not supported.

## General discussion

The hypotheses in the two experiments were generally not supported: No effects of veracity were found with any of the interview techniques, and effects of culture were found sporadically and not always in line with the hypotheses. These findings will be discussed in turn. The fact that we find no veracity effects in these experiments, for any of the interview techniques that were tested, might be a sign that veracity effects in the literature do not generalize to populations outside of those populations in which the cues were originally found. The fact that the lack of veracity effect occurs in participants from both individualistic and collectivistic cultures might suggest that even small changes in the cultural or linguistic background can lead to a failure to generalize results. The current results are broadly in line with Taylor et al. (2014) and Leal et al. (2018) findings that cues to deception differ across cultural populations and that cues uncovered in one cultural context may not readily or directly translate to another. These observations highlight the importance of deception researchers considering cultural factors in the populations that are being studied. Failure to consider cultural factors is a serious limitation to the current literature, and research using a more diverse sample is needed to remedy this problem.

Second, the cultural results for both studies/experiments not only did not support some hypotheses but were directly opposite for others. This observation suggests that the problem with generalizing results from one cultural population to another is not limited to the veracity or lie detection. Rather, it may extend to behaviors that are not necessarily cues to deception. This study is part of a growing body of work that suggests that the effects of culture on verbal and non-verbal behaviors do not generalize robustly. This suggests that the need for more research from currently underrepresented countries and cross-cultural research may extend to the whole of forensic psychology (see, e.g., Hope et al., 2022) and possibly to the whole of psychology (see, e.g., Roberts et al., 2020).

Another finding of interest is that in both experiments, there was a low correlation between participants' COS scores and the Hofstede score of their country of birth. There are several possible explanations for this. Hofstede and Bond (1984) and Triandis and Gelfand (1998) might interpret individualism–collectivism differently: i.e., although they use the same concept of individualism–collectivism, they measure this concept differently and may therefore be inadvertently measuring different concepts. Adding the horizontal–vertical dimension to the COS might have created a measure of a different cultural dimension that does not overlap with Hofstede's individualism–collectivism. The low correlation may also be due to the mismatch between the country level measures that Hofstede and Bond (1984) use and the individual level measures used by Triandis and Gelfand (1998). Voronov and Singer (2002) have suggested that the individualist–collectivist cultural dimension is not sufficiently theoretically developed to be used effectively in psychological research. They argue that large differences in values within countries and methodological concerns surrounding Hofstede's study mean that individualism–collectivism, as measured by the Hofstede score, is often not usable as an independent variable. This may explain the lack of clear effects in these experiments and the literature.

It is also possible that the participants in these experiments, all of whom were migrants to the UK, were unrepresentative of their country of birth. Migration from a collectivist to an individualist country has been associated with changes in cultural identity (Bhugra, 2005) and may lead to changes in cultural values, such as those measured by COS. It is also possible that, for some people, having different values than the country they live in was a cause of the migration. Alternatively, Hofstede scores per country were often collected years before the current experiments, and these studies were not always able to get a fully representative sample of the population of that country (Voronov and Singer, 2002). It may well be that the Hofstede score for certain countries was not or is no longer representative of the countries' cultural values when the participants lived there.

### Limitations

There are a number of limitations to note. First, pertaining to the sample recruited, it is important to note that participants in these experiments were all non-native English-speaking migrants to the UK. The advantage of this sampling approach was that it avoided mixing native with non-native speakers, which could produce a fatal confound reflecting language fluency. However, it has several disadvantages. First, although all participants achieved or self-reported high levels of English, speaking in a second language does affect deception cues (Akehurst et al., 2018; Wylie et al., 2022). Furthermore, non-native speakers use some of the language features of interest differently than native speakers would [e.g., non-native speakers may use “we” more inclusively than native speakers; Dafouz et al. (2007)]. This may affect the cues that were used, particularly in Experiment 1. Second, in Experiment 1, we used LIWC to measure the deception cues: I, we, cognitive processes, and social processes. Although LIWC is regularly used to study the language use of non-native English speakers (e.g., Dhillon et al., 2021), it is possible that non-LIWC-derived deception cues would, unlike LIWC-derived cues, generalize to this sample. Third, both the interview and the COS were conducted in English for all participants. English is spoken in several individualistic countries (e.g., the US and UK) and might act as a prime for an individualistic mind-set and language use that is more associated with individualistic cultures (Lee et al., 2010). Conducting the interviews, in a more culturally neutral second language (e.g., Spanish) may yield different results. Together, these limitations highlight that further research is needed to untangle the effects of culture, language, and how people change as a consequence of migration.

A second limitation is that the research was limited to the individualism–collectivism dimension of culture. Culture is highly variable, has many different aspects, and affects people's behavior in a myriad of ways. Broader measures of culture, such as uncertainty avoidance in a high–low context, might have given us a greater insight into what is making these participants different from participants in previous lie detection studies. It would also be interesting to investigate whether there are cultural differences in the participants' beliefs about deception itself.

Third, the fact that the hypothesized veracity results were not found in the different populations in these experiments does not mean that such veracity results will never be generalizable across populations from different cultures. It may well be that these effects generalize to some cultures, just not the ones tested in these experiments. Conversely, there are only a very small number of experiments showing the robustness of the effects of unexpected questions, sketches, and especially timelines (with only one study) in deception detection. So there may be issues replicating these results even within the same cultural population.

Fourth, the current study focusses on a limited number of dependent variables (four categories of LIWC in Experiment 1; details in Experiment 2). It is possible that other lie detection methods produce cues that generalize across populations.

Fifth, in Experiment 2, due to data loss, the sample was smaller than the power analysis suggested was necessary. Low power might explain the lack of significant results in that study. Further research in this field should ensure sufficient power.

## Conclusion

The results from the two experiments in this study suggest that asking unexpected questions, sketches, and timelines may not be beneficial in eliciting cues to deception in populations outside of those that were tested in the original experiments. The results also suggest that the effects of culture on the behaviors that are used as cues to deception are not always consistent. Overall, we conclude that cultural differences affect our ability to detect lies in ways of which we have only a very limited understanding. More research conducted in countries outside the US and Europe is needed.

## Data availability statement

The raw data supporting the conclusions of this article will be made available by the authors, without undue reservation.

## Ethics statement

The studies involving humans were approved by Faculty of Science and Technology Research Ethics Committee, Lancaster University. The studies were conducted in accordance with the local legislation and institutional requirements. The participants provided their written informed consent to participate in this study.

## Author contributions

IT designed both studies, collected data for both studies, analyzed and drafted study 1, edited the article. LW supervised IT during experimental design and data collected, analyzed and drafted study 2, drafted the general section of the articles, and edited the article. PT and LH supervised IT during experimental design and data collected and edited the article.

## Appendix

**Table A1:** Study 1 question list.

**Question**	**Question type**	**Expectedness rating mean**	**SD**
1. Please describe your intention in as much detail as possible. Please leave nothing out, even if you consider that it might not be important.	Ind > col expected	1.44	0.70
2. How are you going to get to your destination?	All expected	2.02	1.02
3. When traveling to your destination, what part would you say it the most important?	Col > Ind expected	2.73	0.98
4. When you arrive at your destination, who is the first person you will see and why?	All expected	2.40	0.99
5. Have you already done any preparation or planning for this trip?	All expected	1.90	0.92
6. While you were preparing, did you make any alterations to your original travel plan?	Col > Ind expected	2.93	0.91
7. Are you intending to do any preparation or planning for this trip in the future?	Col > Ind expected	2.48	1.04
8. Please list all the people who have something to do with your intention.	Ind > Col expected	2.86	1.02
9. Out of these people, who would you say is the most important person and why?	Ind > Col expected	2.98	1.03
10. Could you please list everyone who you are leaving behind during your trip.	All unexpected	3.55	0.72
11. Who is the most important person who is staying behind and why?	All unexpected	3.39	0.83
12. How will your trip affect the people you are leaving behind?	All unexpected	3.52	0.76
